# ﻿A new species of *Pleurothallis* (Pleurothallidinae, Orchidaceae) from the historic sanctuary of Machupicchu, Perú

**DOI:** 10.3897/phytokeys.254.142116

**Published:** 2025-03-20

**Authors:** Alexander Damián-Parizaca, Marco Federico Monteros, Daxs Coayla Rimachi, Joseph Walston, Nicole Mitidieri-Rivera

**Affiliations:** 1 Department of Botany, University of Wisconsin-Madison, 430 Lincoln Drive, Madison, Wisconsin, USA Inkaterra Asociacion Victor Larco Herrera Lima Peru; 2 Inkaterra Asociacion Victor Larco Herrera, 130 Miraflores, Lima, Peru University of Wisconsin-Madison Madison United States of America; 3 Fundacion EcoMinga, Mariscal Foch 7-21 y Juan León Mera, Quito, Ecuador Fundacion EcoMinga Quito Ecuador; 4 Reserva: The Youth Land Trust, Washington, D.C., USA Reserva: The Youth Land Trust Washington United States of America; 5 Instituto Nacional de Biodiversidad (INABIO), Rumipamba 341 y Av. De los Shyris, Quito, Ecuador Instituto Nacional de Biodiversidad Quito Ecuador; 6 Grupo Científico Calaway Dodson: Investigación y Conservación de Orquídeas del Ecuador, Quito, 170510, Pichincha, Ecuador Grupo Científico Calaway Dodson: Investigación y Conservación de Orquídeas del Ecuador Quito Ecuador

**Keywords:** Andes, biodiversity, Cusco, Inkaterra

## Abstract

*Pleurothallismachupicchuensis*, a new species from Cusco, Perú, is described and illustrated. Information regarding its distribution, habitat, and phenology is provided. Morphologically, *P.machupicchuensis* closely resembles *P.scurrula* and *P.sannio* but can be distinguished mainly by the morphology of the lip. The new species features an ovate, obtuse lip with a prominent bilobed, reniform glenion, in contrast to the oblong-ovate, acute lip with a small rounded glenion observed in *P.scurrula*, and the ovate, rounded lip with an oblong glenion in *P.sannio*.

## ﻿Introduction

The genus *Pleurothallis* R. Br. is among the largest groups within the subtribe Pleurothallidinae Lindl. comprising nearly 500 recognized species, with new taxa being described regularly (e.g. Karremans and Vieira-Uribe 2020). Classifying such a megadiverse group has posed significant challenges to numerous botanists, including Lindley (1842, 1859) and Luer (1998, 1999, 2005), who proposed the earliest and most recent morphology-based classification systems of *Pleurothallis*. As noted by Karremans (2016), both Lindley and Luer acknowledged the limitations of their classifications, recognizing the absence of more satisfactory solutions given the available data. A major breakthrough in the classification of *Pleurothallis* was achieved with the work of Pridgeon and colleagues (Pridgeon et al. 2001), who introduced the first DNA-based classification system for Pleurothallidinae. This molecular approach demonstrated that a natural classification of *Pleurothallis* was attainable, although it required extensive modifications to Luer’s circumscription. Subsequent revisions, initiated by Pridgeon himself (Pridgeon and Chase 2001) and expanded upon by various authors over the past two decades (e.g. Karremans et al. 2013; Karremans 2019), have successfully established the monophyly of *Pleurothallis* by refining its circumscription. These efforts have also clarified its phylogenetic placement within a well-supported clade that includes *Pabstiella* and *Stelis*. However, the infrageneric relationships within *Pleurothallis* remain largely unresolved (Pridgeon 2005; Karremans 2016).

One of the most challenging groups to circumscribe within *Pleurothallis* is Pleurothallissect.Macrophyllae-Fasciculatae Lindl. Originally established by Lindley (1859) to include species characterized by terete-angulate stems, cordate leaves, and fasciculate flowers, this section was later incorporated into a broader circumscription as a section within *Acronia* C. Presl by Luer (2005). Recent phylogenetic studies utilizing Sanger-based DNA sequencing have demonstrated that species assigned to Luer’s *Acronia* are distributed across multiple clades, indicating its non-monophyly (Wilson 2011; 2013; Pridgeon et al. 2011). These findings have also uncovered a highly intricate evolutionary history, shaped by its recent diversification (~5 Ma) and natural hybridization events within the group (Pérez-Escobar et al. 2017; Pupulin et al. 2021). Given the absence of a robust phylogenetic framework for *Pleurothallis*, recent authors have increasingly treated Pleurothallissect.Macrophyllae-Fasciculatae as an informal assemblage or a collection of morphological complexes rather than a monophyletic, phylogenetically well-defined group (Pupulin et al. 2021; Wilson et al. 2022).

Although *Macrophyllae-Fasciculatae* continues to be referenced in the literature, either at the subsectional (e.g., Baquero et al. 2024; Sierra-Ariza 2024) or sectional (Belfort-Oconitrillo et al. 2024) level within *Pleurothallis*, we concur with previous authors that it is more appropriate to recognize this group as an assemblage of species complexes with poorly defined boundaries. Accordingly, in this manuscript, we adopt the definition proposed by Pupulin et al. (2021), who described members previously assigned to Pleurothallissect.Macrophyllae-Fasciculatae as a group of *Pleurothallis* species distinguished by their tall growth habit and fasciculate inflorescences borne above the leaf from a spathaceous, occasionally erect bract. However, it is crucial to point out that recent analyses have questioned the suitability of the term “fasciculate” in describing the inflorescences of this group, suggesting instead that these structures are more accurately interpreted as single-flowered co-florescences (Rojas-Alvarado and Karremans 2024).

The absence of a well-defined circumscription presents a significant challenge in assessing the true diversity of this group. However, previous estimates suggest it comprises approximately 300 species endemic to the Neotropics, occupying a broad range of biomes and ecosystems, from sea level to elevations exceeding 3,000 meters (Pridgeon 2005; Wilson et al. 2022). Despite its extensive distribution, the highest species diversity is believed to occur in South America, particularly in the Central and Northern Andes. This is reflected in the frequent discovery of new species and records from Andean countries (e.g., Jiménez et al. 2023; Ocupa-Horna et al. 2023a). While some regions, such as Colombia (Karremans et al. 2023), have recently published comprehensive or updated assessments of Pleurothallidinae diversity, Perú still lacks a modern taxonomic assessment for this group. For instance, Luer’s monograph on Acroniasect.Macrophyllae-Fasciculatae (Lindl.) Luer recorded approximately 30 species for Perú (Luer 2005). However, as demonstrated in other orchid genera native to the country (e.g., *Lepanthes* [Ocupa-Horna et al. 2023b]; *Acianthera* [Damián-Parizaca et al. 2018]), this estimate is likely incomplete. Ongoing field exploration and taxonomic research are anticipated to reveal many additional species.

One of Perú’s most orchid-rich sites is the Historic Sanctuary of Machu Picchu (SHM), a protected area encompassing approximately 30,000 hectares and home to around 400 orchid species (Collantes et al. 2007). This diversity continues to be revised, with new species reported regularly (e.g., *Stelismachupicchuensis*, Collantes et al. 2017; *Telipogonmachupicchuensis*, Nauray and Christenson 2003). During the 7^th^ Scientific Conference on Andean Orchids, held in Machupicchu Pueblo in November 2024, one of the authors (MM) observed an undescribed *Pleurothallis* species growing in the garden of the Inkaterra Machu Picchu Pueblo Hotel. This species is formally described herein, along with a detailed comparison to its closest morphological relatives.

## ﻿Materials and methods

To assess whether the plant observed at the Inkaterra Machupicchu Pueblo Hotel represents an undescribed species, we conducted a detailed morphological analysis. Initially, we compared *Pleurothallis* taxa previously described and recorded in Perú, subsequently expanding our assessment to include other Andean taxa. Given the absence of an updated taxonomic treatment of species formerly assigned to Pleurothallissect.Macrophyllae-Fasciculatae we relied primarily on Luer’s monograph of *Acronia*, supplementing it with a review of recently described *Pleurothallis* species published post-Luer. Recognizing that vegetative traits often provide limited diagnostic utility for distinguishing closely related species, we focused our analysis on floral characteristics. Morphological descriptions follow the framework of Wilson et al. (2022) complemented by the updated inflorescence terminology for Pleurothallidinae proposed by Rojas-Alvarado and Karremans (2024). Additionally, we conducted a preliminary herbarium review using the Tropicos.org, Global Biodiversity Information Facility (GBIF), and Atrium databases. Many herbarium specimens lacked flowers or were too small to exhibit diagnostic traits aligning with our concept of the proposed new species. A physical examination of the CUZ herbarium similarly failed to uncover any specimens resembling *P.machupicchuensis* inedit.

To supplement our analysis, we searched for live photographs of *Pleurothallis* across several databases, including GBIF, iNaturalist, Flickr, and Atrium. When available, the metadata accompanying these photographs was collected and incorporated into our downstream analysis. Distribution maps were generated in R Studio using a modified script from Damián-Parizaca and Mitidieri-Rivera (2023). For specimens lacking coordinates, we approximated collection locations based on label or protologue information. The final maps were edited in Adobe Photoshop® v.22.5.1. Line drawings, including both line work and stippling, were produced from photographs of dissected perianths using a Wacom Intuos Wireless Graphics Tablet in Adobe Photoshop CS6.v13. Additionally, a Lankester Composite Dissection Plate (LCDP) was created from macro photographs taken with a Nikon D810 camera, and the images were subsequently edited and arranged in Adobe Photoshop.

To further investigate labellum morphology, we used Scanning Electron Microscopy (SEM). Two flowers were dehydrated through a stepwise transition from 70% to 100% ethanol, with fifteen-minute increments at 80%, 95%, and 100%, followed by critical-point drying with a Leica EM CPD300. The samples were mounted onto aluminium SEM stubs, sputter-coated with platinum-palladium using a Leica EM ACE600, and imaged with a FEI Quanta 200 scanning electron microscope at 20 kV accelerating voltage and a 10–12 mm working distance. SEM imaging was conducted at the Newcomb Imaging Center, Department of Botany, University of Wisconsin–Madison.

## ﻿Results

### ﻿Taxonomic treatment

#### ﻿ Pleurothallis machupicchuensis

Taxon classificationPlantaeAsparagalesOrchidaceae

Damián-Parizaca, Monteros & Coayla sp. nov.

610ED54E-1FE8-5EAF-A7B6-28F83DBFF019

urn:lsid:ipni.org:names:77358955-1

Figs 1 , 2 , 3A , 4

##### Type.

Perú • Cusco, Prov. Urubamba, Aguas Calientes, property of the Machupicchu Pueblo Hotel, 2000 m, 30 November 2023, *Daxs Coayla 001* (***holotype***: CUZ).

##### Diagnosis.

*Pleurothallismachupicchuensis* is most similar to *Pleurothallisscurrula* Luer but differs by the obtuse dorsal sepal (vs. acute), the acute synsepal apex (vs. obtuse to rounded), the falcate petals (vs. oblong-ovate), the ovate lip (vs. oblong), and the reniform bilobed glenion (vs. oblong non bilobed).

##### Description.

***Plant*** epiphytic, caespitose, erect 15 cm tall. ***Roots*** slender, flexuose, up to 0.1 cm in diameter. ***Ramicauls*** erect, 10–15 cm long, 0.1 cm in diameter, slightly curved at the apex forming an angle of about 100° in the abscission layer, enclosed by two basal papyraceous, sulcate, brownish, tubular sheaths, 4.0–6.5 cm long. ***Leaf*** borne at the apex of the ramicaul, suberect to nearly horizontal, pale green, slightly coriaceous, deflexed toward the base, somewhat concave, lanceolate, with a short concavity at the base of the spathe, margins entire, acuminate, 5.0–7.7 × 2.0–3.0 cm, base sessile, cordate, lobes equal. ***Inflorescence*** a single-flowered coflorescence borne erect from a depressed, conduplicate, oblong, obtuse, sub-erect spathe at the base of the leaf, 0.8–1.0 cm long, striate, brownish, dry-papyraceous when mature, concealing peduncle, branch system, pseudopeduncle and pedicel; ***pseudopeduncle*** terete, up to 0.4 cm long; ***pedicel*** yellowish, flexuose, terete, 1.0–1.5 cm long; ***ovary*** terete, blackish to brownish, furrowed, 0.5–0.7 cm long. ***Flowers*** non-resupinate, spreading, yellowish to citrine colored, column yellowish to whitish, anther cap and stigma yellowish, lip overall yellowish sometimes with reddish margins. ***Dorsal sepal*** erect slightly convex, ovate, glabrous, obtuse, 3-veined, 1.0 × 0.5 cm. ***Lateral sepals*** connate into a broadly ovate synsepal, centrally concave-channeled, obtuse, glabrous, margins entire, 0.8 × 0.6–0.7 cm, 4-veined. ***Petals*** strongly reflexed, falcate, 1-veined, conspicuously papillate on the margins, 0.6–0.7 × 0.1 cm. ***Labellum*** overall ovate, obtuse at the apex, slightly ascending, strongly verrucose-bullate towards the margins, papillae grouped into clumps of around 5–6 papillae, clumps join together to form nearly horizontal linear groups that follow down to the base, centrally sulcate, base acutely deflexed upon itself hinged to the column-foot, 0.3 × 0.2 cm; ***glenion*** bilobed, hourglass-shaped, ca. 400 µm wide, ca. 150 µm at its shortest, and ca. 250 µm at its longest length, papillae at the glenion boundary smooth and notably larger and taller, width 25 µm and length 35 µm, papillae found outside of the glenion are highly textured, width 25 µm. ***Column*** short, stout, complanate, 0.2 cm long, minutely papilose, rostellar flap long, linear, obtuse. ***Anther*** apical, incumbent, anther cap cucullate, ovate, 2-celled, 0.8 × 0.6 mm. ***Stigma*** apical, bilobed, reniform. ***Pollinia*** two, narrowly pyriform, 0.1 cm long attached to an elliptic viscidium. ***Fruit*** unknown.

##### Phenology.

This species has been observed flowering in two seasons: April and from October to December.

##### Etymology.

The epithet honors the *llaqta* Machupicchu, an Inka citadel in Cusco, southern Peru, located within the Urubamba Province, where *P.machupicchuensis* is locally distributed.

##### Distribution and habitat.

*Pleurothallismachupicchuensis* primarily grows on trees of the genus *Clusia* L. within typical montane forest vegetation. Known populations are located along the Urubamba and Usmubamba Rivers in the provinces of Urubamba and La Convención, respectively, as well as within the Machu Picchu Historic Sanctuary, at elevations ranging from 2000 to 2500 meters (Fig. 2).

##### Conservation status.

Although the known distribution of *Pleurothallismachupicchuensis* is within the Machu Picchu Sanctuary, sufficient information is still lacking to assess its preliminary conservation status. Therefore, we recommend classifying this species as Data Deficient (DD) according to the IUCN (2022) criteria.

##### Taxonomic notes.

**Figure 1.**
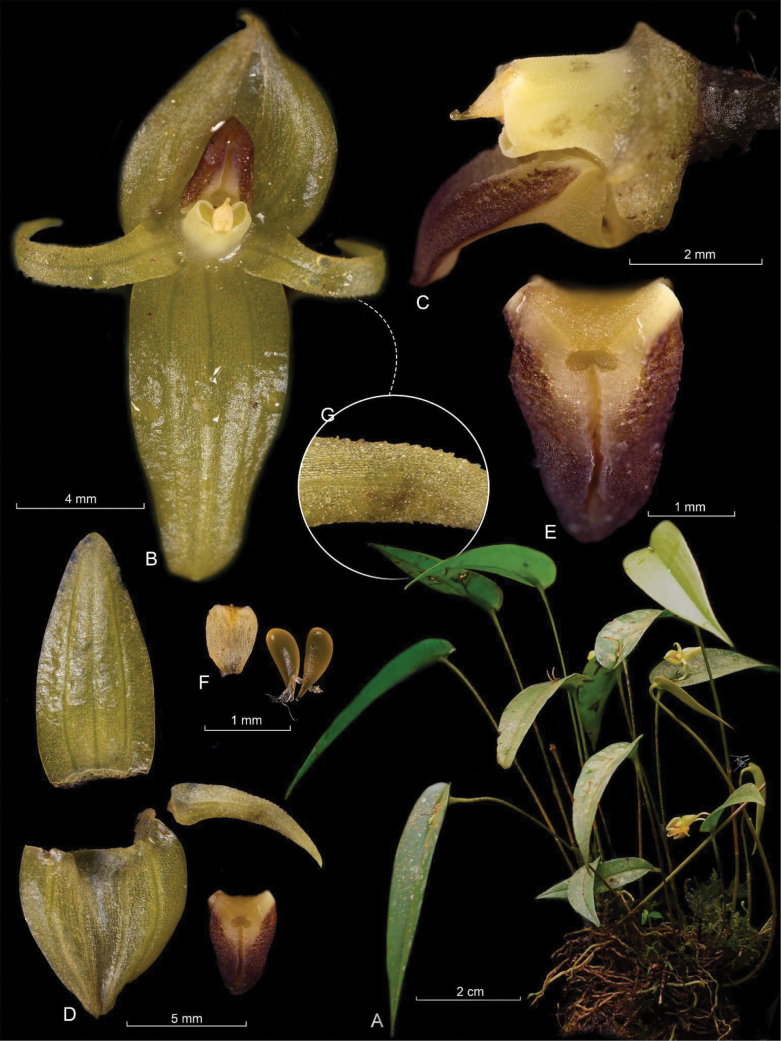
Composite digital plate of *Pleurothallismachupicchuensis***A** habit **B** flower **C** lip and column in lateral view **D** dissected flower **E** lip adaxial view **F** anther and pollinia **G** petal margin. Prepared from the holotype by Alexander Damián-Parizaca.

**Figure 2.**
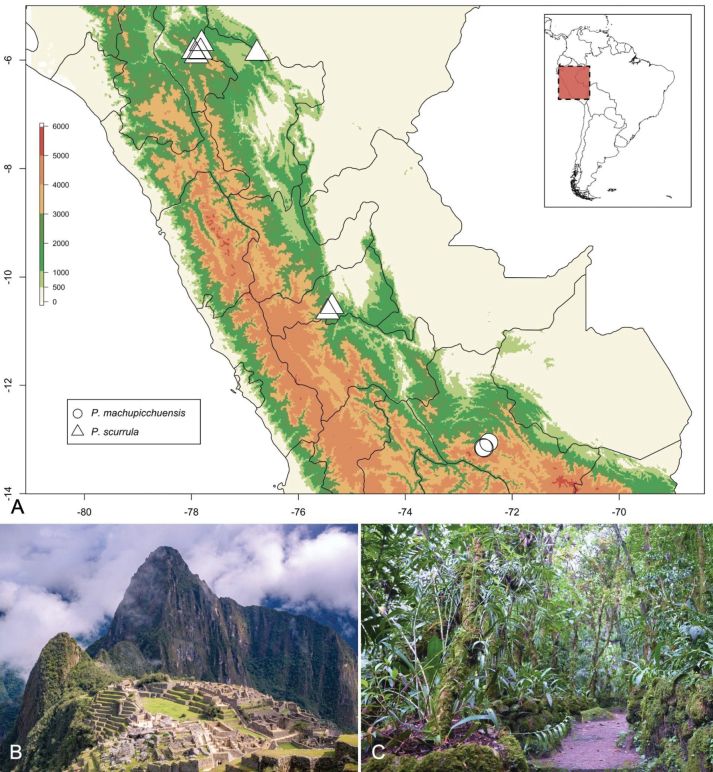
**A** map of known localities of *Pleurothallismachupicchuensis* and *Pleurothallisscurrula***B** panoramic view of the Machu Picchu Historical Sanctuary **C** Inkaterra Machu Picchu Pueblo Hotel Garden. Photographs by (**B**) Inkaterra, (**C**) Daxs Coayla.

**Figure 3.**
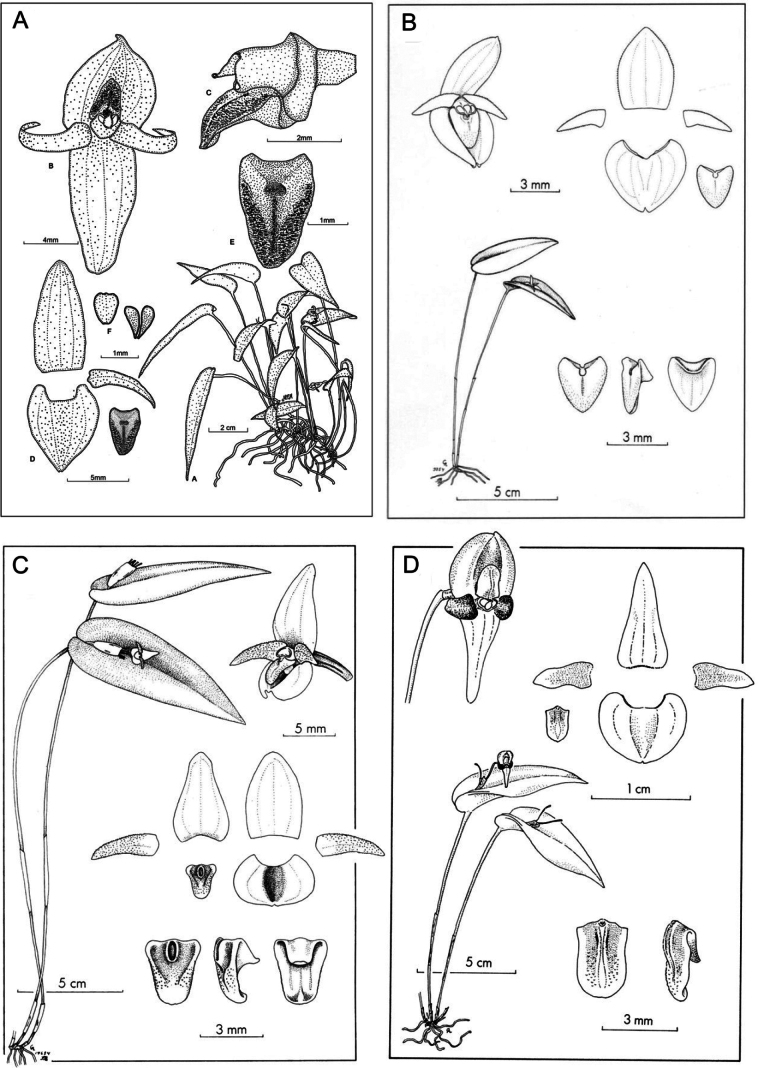
Line drawings of the species treated in the present study **A***P.machupicchuensis* prepared by MM based on the holotype **B***P.phyllocardioides* (=*P.graciliscapa*) **C***P.sannio***D***P.scurrula*. (**B–D**) from Luer (2005) courtesy of the Missouri Botanic Gardens Press.

*Pleurothallismachupicchuensis* is readily distinguished by the combination of a large dorsal sepal (relative to the synsepal), non-resupinate flowers, falcate petals, and an ovate lip that is conspicuously sulcate and features a prominent bilobed glenion (Figs 1, 2A). The species was first photographed in 1998 at the Inkaterra Pueblo Hotel and has since been mistakenly identified as *Pleurothallisphyllocardioides* Schltr. (Collantes et al. 2007; Ochoa 2023). Luer (2005) recognized *P.phyllocardioides* as part of a species complex widely distributed from Central America throughout the Andes. This complex is characterized by having short and non-reflexed petals measuring 2–3 mm, and an ovate-oblong lip with an unlobed glenion. These features differ significantly from strongly reflexed petals measuring 6–7 mm, and the ovate lip with a bilobed glenion observed in *P.machupicchuensis* (Fig. 3A, B). Furthermore, Andean populations of *P.phyllocardioides* reported by Luer (2005) are found in submontane forests below 1200 m altitude, whereas *P.machupicchuensis* has so far been documented exclusively in montane forests above 2000 m. Additionally, *P.phyllocardioides* appears to be restricted to Central America (M. Wilson, pers. comm.), and Peruvian populations previously described as *Pleurothallisgraciliscapa* C. Schweinf. (= *P.phyllocardioides* sensu Luer 2005) show minimal divergence from the typical Central American morphotype, rendering their resemblance to *P.machupicchuensis* less plausible.

**Figure 4.**
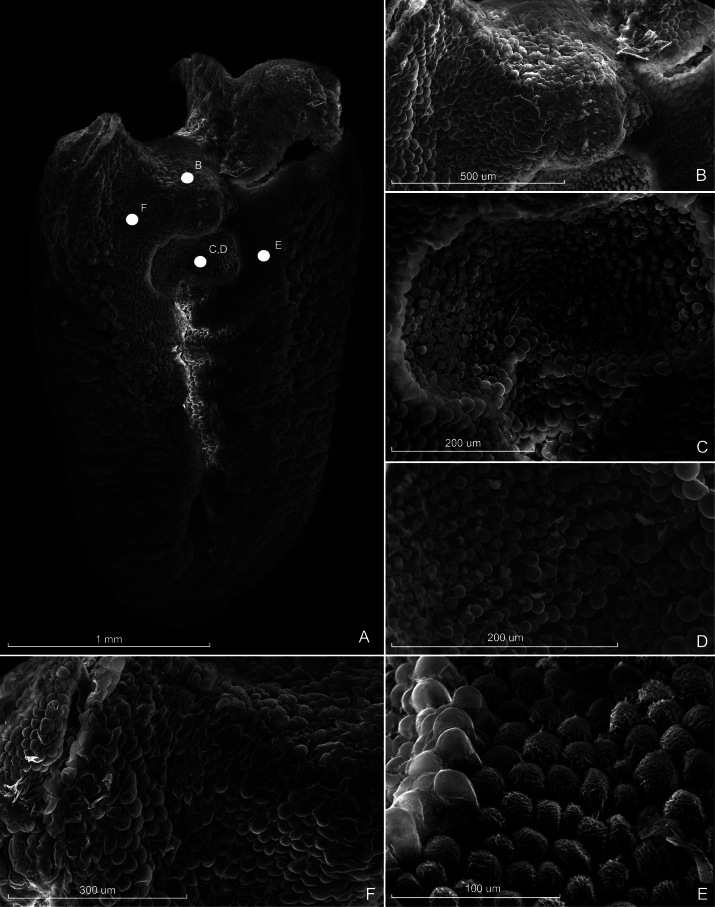
Scanning electron micrographs of *Pleurothallismachupicchuensis* lip **A** general view of the lip **B** detail close-up of the superior part of the lip which overlaps with the column **C, D** glenion **E, F** lateral sides of the lip. Prepared from holotype *D. Coayla 001* (CUZ) by Joseph Walston.

The Peruvian *Pleurothallisscurrula* is the most morphologically similar species to *P.machupicchuensis* but can be readily distinguished, primarily by its petal shape. In *P.scurrula*, the petals are oblong-ovate, whereas in *P.machupicchuensis*, they are conspicuously falcate. The two species are also differentiated by the morphology of the labellum, which is clearly rounded in *P.scurrula* and obtuse in *P.machupicchuensis*. Furthermore, *P.machupicchuensis* possesses a conspicuous bilobed glenion, contrasting with the minute, rounded glenion of *P.scurrula*. Another morphologically similar species is the Colombian *Pleurothallissannio* Luer & R. Escobar, which can be distinguished by its slightly shorter flowers, with sepals measuring 8 mm in length (versus 10 mm in *P.machupicchuensis*), and its obovate synsepal (versus ovate). Most notably, *P.sannio* lacks a bilobed glenion and a central furrow on the lip, features that are diagnostic of *P.machupicchuensis* (Table 1, Fig. 3C).

**Table 1.** Morphological comparison of *Pleurothallis* species morphologically similar to *P.machupicchuensis*.

	*P.machupicchuensis*	*P.sannio*	*P.scurrula*
Leaf shape	lanceolate, apex acuminate	narrowly ovate, apex acute	ovate, apex acute
Leaf dimensions (cm)	5–7.7 × 2–3	9–10 × 2.5–3 cm	5–8 × 2–3
Spathe dimensions (cm)	0.8–1.0	1.5	1–1.5
Dorsal sepal shape	ovate, apex obtuse	ovate, apex obtuse	ovate-triangular, apex acute
Dorsal sepal dimensions (mm)	10 × 5	8 × 5.5	7 × 9
Synsepal shape	ovate, apex obtuse	obovate	ovate, apex rounded
Synsepal-dimensions (mm)	8 × 6–7	8 × 4	7 × 9
Petals shape	falcate, apex acuminate	obliquely triangular-ovate, apex acute	oblong-ovate, apex acute
Petals dimensions (mm)	6–7 × 1	6 × 2	7 × 3
Lip shape	ovate, apex obtuse	ovate, apex rounded	oblong-ovate, apex rounded
Lip dimensions (mm)	3 × 2	3 × 2.5	3 × 2
Glenion	hourglass-shaped, bilobed	oblong	rounded

##### Other records.

Perú • Cusco, Prov. Urubamba, Aguas Calientes, Catarata de Mandor, December 20, 2021 [flower] https://www.inaturalist.org/observations/103258849; • ibid. January 5, 2013, [flower] https://www.inaturalist.org/observations/96696628; • Prov. La Convencion, Usmabamba, February 10, 2020 [flower] https://www.inaturalist.org/observations/68871779; • Prov. Urubamba, Machu Picchu Historic Sanctuary, 1998, reported by the Inkaterra Association Research Team, pl. 114 as *Pleurothallisphyllocardioides* Schlechter (Collantes et al. 2007); • Machupicchu Historical Sanctuary, at 1900–2500 m, March 24, 2012 [flower] https://bit.ly/3WtKfEB; • Cusco, Aguas Calientes, 26 April 2010 [flower] https://bit.ly/4ao1Vak.

## Supplementary Material

XML Treatment for Pleurothallis machupicchuensis

## ﻿Additional information

### Author ORCIDs

Alexander Damián-Parizaca https://orcid.org/0000-0002-0233-9935

Marco Federico Monteros https://orcid.org/0000-0002-6670-3687

Joseph Walston https://orcid.org/0009-0007-1702-8703

Nicole Mitidieri-Rivera https://orcid.org/0000-0001-6045-4491

### Data availability

All of the data that support the findings of this study are available in the main text.
